# Supplementary material for: Histone deacetylation promotes transcriptional silencing at facultative heterochromatin
Source: Nucleic Acids Res. 2018 Mar 29;46(11):5426–40. doi: 10.1093/nar/gky232 (PMC6009587; doi:10.1093/nar/gky232)
Supplement: Supplementary Data [file gky232_supplemental_files.zip › Supplementary Figures and Tables Legends.pdf]

Supplementary Figures and Tables Legends:

Figure Supplement 1: (a) Schematic of the *pho1* gene is shown with probe indicated by a black bar (top). Northern blot analysis of RNA isolated from the indicated strains grown in YES. 18S ribosomal RNA and *adh1* serve as loading controls.

Figure Supplement 2: Schematic of the *pho1* gene is shown with qPCR products indicated by black bars. Mmi1-HTP (a) and Pol II (b) ChIP-qPCR analysis over the *pho1* locus either untreated (-) or treated (+) with RNase.

Figure Supplement 3: (a) Schematic of *tgp1* locus with probe indicated (top). Northern blot analysis of *tgp1* levels from RNA isolated from the indicated strains in the presence (+) or absence (-) (for either 12 or 24 hours) of 10 mM KH_2PO_4. 18S ribosomal RNA serves as a loading control. (b) Northern blot analysis of RNA isolated from a wild-type (WT) strain untreated (-TSA) or treated (+TSA) with 20 µg/mL of the HDAC inhibitor trichostatin A in the presence (+) or absence (-) of 10 mM KH_2PO_4. *adh1* serves as a loading control. The *tgp1* probe used is as in (a).

Figure Supplement 4: (a) Differential expression plots for RNA-seq (left) and NET-seq (right) datasets. (b) Spearman correlation matrix between all biological replicates of all strains that were used in H3K14ac ChIP-seq experiments. Bam alignment files were used to calculate the Spearman correlation coefficient after dividing the genome in 200 nt bins, using deepTools version 2.2.2. (c) Metagene plot to identify the peak region of H3K14ac. Two replicates of acetylation profiles +/- TSA in the region 500 nt before TSS to 500 nt after the TES are shown. The region 75 nt before to 300 nt after TSS was used for comparison with RNA-seq (Figure 4c). (d) Differential acetylation plot for H3K14ac ChIP-seq.

Figure Supplement 5: Schematics of the *mel1/hry1* locus (i), meiotic genes *mug147* (ii), and *mei2* (iii), the calcium/potassium exporter *cta3* (iv), and *SPAC27D7.09c/11c* (v) with H3K14ac ChIP-seq signal (blue) and RNA-seq reads (red) +/- TSA visualized using IGB.

Figure Supplement 6: Schematics of the *ght* hexose transmembrane transporters *ght8* (i), *ght5* (ii), *ght6* (iii), *ght1* (iv), and selfish pseudogenes *wtf3/4* (v), *wtf9* (vi), *wtf11* (vii), and *wtf21* (viii) loci with H3K14ac ChIP-seq signal (blue) and RNA-seq reads (red) +/- TSA visualized using IGB.

Figure Supplement 7: (a) Heatmap view of RNA-Seq signal for CUTs (n = 2509) in WT and *rrp6Δ* cells. (b) Scatter plot of tag density for protein-coding genes (grey) and CUTs (red) in WT and *rrp6*Δ, computed using uniquely mapped reads. Results are presented as $\log_2$ of density, expressed in tag/nt. The black dashed line indicates no change (*rrp6*Δ/WT ratio = 1). (c) Box-plot of *rrp6*Δ/WT RNA-Seq signal ratio for protein-coding genes, snoRNAs, CUTs, ncRNAs (from Pombase.com), novel ncTUs (75) and XUTs.

Supplementary File 1

Supplementary Table 1: RNA-, NET-, and ChIP-seq datasets

Supplementary Table 2: GO analysis of genes with increased transcription and H3K14ac

Supplementary Table 3: List and coordinates of CUTs

Supplementary Table 4: List of CUTs overlapping ORFs with associated log2 fold change in NET-seq and H3K14ac ChIP-seq

Supplementary Table 5: List of *S. pombe* strains used in this study.

Supplementary Table 6: List of *S. pombe* oligonucleotides used in this study.
